# Sunitinib for the treatment of benign and malignant neoplasms from von Hippel-Lindau disease: A single-arm, prospective phase II clinical study from the PREDIR group

**DOI:** 10.18632/oncotarget.13301

**Published:** 2016-11-11

**Authors:** Stéphane Oudard, Reza Elaidi, Mara Brizard, Céline Le Rest, Valérie Caillet, Sophie Deveaux, Gérard Benoit, Jean-Michel Corréas, Farida Benoudiba, Philippe David, Alain Gaudric, Pascal Hammel, Dominique Joly, Marc Olivier Timsit, Arnaud Méjean, Stéphane Richard

**Affiliations:** ^1^ Department of Medical Oncology, Hôpital Européen Georges Pompidou, AP-HP, Paris, France; ^2^ Réseau Expert National pour Cancers Rares de l’Adulte PREDIR AP-HP/INCa, Hôpital Bicêtre, Le Kremlin Bicêtre, France; ^3^ Association pour la Recherche sur les Thérapeutiques Innovantes en Cancérologie, HEGP, Paris, France; ^4^ Department of Ophthalmology, Hôpital Lariboisière, Paris, AP-HP, France; ^5^ Department of Urology, Hôpital Bicêtre, AP-HP, Le Kremlin-Bicêtre, France; ^6^ Department of Radiology, Hôpital Necker, AP-HP, Paris, France; ^7^ Department of Neuroradiology, Hôpital Bicêtre, Le Kremlin-Bicêtre, AP-HP, France; ^8^ Department of Neurosurgery, Hôpital Bicêtre, Le Kremlin-Bicêtre, AP-HP, France; ^9^ Department of Digestive Oncology, Hôpital Beaujon, Clichy, AP-HP, France; ^10^ Department of Nephrology, Hôpital Necker, Paris, AP-HP, France; ^11^ Department of Urology, Hôpital Européen Georges Pompidou, Paris, AP-HP, France; ^12^ Ecole Pratique des Hautes Etudes, Paris, France Laboratoire de Génétique Oncologique EPHE, INSERM, Villejuif, France and Faculté de Médecine Université Paris-Sud, Le Kremlin-Bicêtre, France

**Keywords:** sunitinib, von Hippel-Lindau disease, renal cell carcinoma, hemangioblastoma, toxicity

## Abstract

Von Hippel-Lindau (VHL) disease is an autosomal dominant hereditary cancer syndrome that predisposes affected individuals to the development of multiple benign and malignant tumors. One of the main manifestations of VHL is renal cell carcinoma (RCC). RCC is increasingly being treated with targeted therapies, which offer an alternative treatment option for patients with VHL disease. This study investigated the effectiveness of sunitinib in VHL patients with advanced tumors or tumors unsuitable for surgery.

This multicenter, phase II, open-label study from the PREDIR VHL network, treated patients with genetically-confirmed advanced VHL disease with oral sunitinib (50 mg/day for 28 days then a 2-week rest period) until progression. Lesions were performed using magnetic resonance imaging (MRI) and computed tomographic (CT) scan. The primary endpoint was objective response rate; secondary endpoints included tolerability and overall survival.

All five patients showed stable disease as best response at 6 months. Two patients showed impressive transitory clinical improvement during early cycles. No patient died during sunitinib treatment. Reasons for discontinuing sunitinib therapy were disease progression (n=1), unacceptable toxicity (n=3) and lack of clinical improvement after 7 cycles (10.5 months) with unacceptable toxicity (n=1).

In conclusion, sunitinib was of limited benefit in patients with advanced VHL disease, but had better efficacy against metastatic RCC than other VHL-related lesions. Treatment-related toxicity is an important limiting factor in this frail patient population. New agents with different mechanisms of action are required to treat this disease.

## INTRODUCTION

Von Hippel-Lindau (VHL) disease is a serious, autosomal dominant, neoplastic syndrome that increases susceptibility to a variety of benign and malignant neoplasms. Clinical manifestations of VHL include central nervous system (CNS) and retinal hemangioblastomas, endolymphatic sac tumors, clear-cell RCC, phechromocytomas and pancreatic neuroendocrine tumors. The main cause of death is metastatic RCC (mRCC), but other tumors can be life-threatening or are associated with significant morbidity.

The advent of targeted therapies, including sunitinib and other vascular endothelial growth factor (VEGF) receptor-targeted agents (e.g. axitinib, sorafenib, and pazopanib) and the anti-VEGF antibody bevacizumab, has significantly improved disease-specific survival in mRCC patients [1]. The mammalian target of rapamycin (mTOR) inhibitors, everolimus and temsirolimus, have also become therapeutic options for the treatment of mRCC [2]. Similarly, sunitinib and everolimus are currently indicated for the treatment of patients with sporadic pancreatic neuroendocrine tumors [3–5].

Sunitinib has been shown to be beneficial after cytokine failure in sporadic mRCC [6], and may also be an alternative treatment option for patients with VHL disease. Promising results have been reported in VHL patients treated with semaxanib (a discontinued anti-VEGF tyrosine kinase inhibitor). One patient with refractory retinal hemangioblastomas had a marked improvement in visual acuity after 7 months’ semaxinib treatment [7], another with RCC likely due to VHL had a complete metabolic and radiological response that persisted for 18 months after treatment [8], and a the response rate in a small clinical trial (n=6) was 33% [9]. Preliminary experience with sunitinib in VHL is also promising; six months’ treatment in a patient with multiple renal and pancreatic tumors and a malignant pheochromocytoma was associated with normalization of performance status, pain resolution, and reductions in tumor size [10], another patient with VHL-related mRCC achieved complete remission of these tumors after sunitinib therapy [11], and partial response/stable disease was evident in a patient with pancreatic neuroendocrine and RCC tumors who received sunitinib for nearly 5 years [12].

This study investigated the effectiveness of sunitinib in VHL patients with advanced tumors or tumors unsuitable for treatment with other available options (surgery or laser).

## RESULTS

### Patients

We identified 354 patients who were being followed up for VHL disease in the PREDIR network over the 1-year study period, and planned to enroll 20 patients. However, only a very small proportion of these was not eligible for further focal treatment, and an even smaller subgroup agreed to participate in our study. Therefore, the accrual rate was low and recruitment was stopped after screening of 7 patients. Of these, five had genetic confirmation of VHL disease, met all the eligibility criteria and were enrolled. Patient demographic and clinical characteristics at baseline are shown in Table 1. A total of 6 target lesions and 8 non-target lesions were present in the 5 study patients. Three patients (60%) had target lesions (maximum of five target lesions in one patient). The smallest target lesion was 10 mm and the largest was 44 mm. Three patients (60%) had non-target lesions (maximum of five non-target lesions in one patient).

**Table 1:** Patient demographic and clinical characteristics at baseline

	Patients (n=5)
Age, years; median (range)	65 (52-81)
Male, n (%)	4 (80)
VHL disease manifestation, n (%):	
Renal cell carcinoma	3 (60)
CNS hemangioblastomas	5 (100)
Retinal hemangioblastomas	3 (60)
Pancreatic cysts	3 (60)
Pancreatic neuroendocrine tumor	0
Pheochromocytoma	0
Endolymphatic sac tumor	0
Epididymal cystadenoma	1 (20)
VHL mutation, n (%):	
c.C211A, 211_212insA	1 (20)
c.217C>G, p.Gln73X	1 (20)
c.256G>T, p.Pro86Ser	1 (20)
c.345C>G, p.His115Gln	1 (20)
1-?,_642+?del, complete deletion	1 (20)
ECOG-PS, n (%):	
0	3 (60)
1	2 (40)
LVEF, %	58.5 (50-70)
Hemoglobin, g/dL	15.2 (12.9-17.8)
Bilirubin, μg/L	13.6 (8.5-19)
ALT, IU/L	26.2 (11-55)
AST, IU/L	40.2 (13-81)
LDH, IU/L	188 (140-225)
Alkaline phosphatases, IU/L	131 (37-1252)
Type of progression at study entry, n:	
Imaging only	0
Imaging + clinical	5
Clinical only	0

Values are median (range) or number of patients.

ALT, alanine aminotransferase; ALT, alanine aminotransferase; ECOG-PS, European Co-operative Oncology Group Performance Status; LDH, lactate dehydrogenase; LVEF, left ventricular ejection fraction; VHL, von Hippel-Lindau.

### Sunitinib treatment

All patients were started on sunitinib 50 mg/day (schedule 4/2). Drug exposure and dose intensity is summarized in Table 2.

**Table 2:** Sunitinib exposure and dose intensity

Sunitinib (schedule 4/2)	Number of treated patients	Patients requiring dose reduction
Cycle 1	5	1*
Cycle 2	5	2**
Cycle 3	5	1*
Cycle 4	3	0
Cycle 5	1	0
Cycle 6	1	0
Cycle 7	1	0
Cycle >7	0	0

* Dose reduced to 37.5 mg/day.

** Dose reduced to 37.5 mg/day in one patient, and to 37.5 mg/day then to 25 mg/day in one patient.

### Overall response rate

The best clinical responses were observed with sunitinib 50 mg/day (schedule 4/2). All patients showed stable disease (based on imaging) at 6 months, apart from severe worsening of retinal hemangioblastoma tumors in one patient (whose tumors at other locations remained stable).

**Figure 1:**
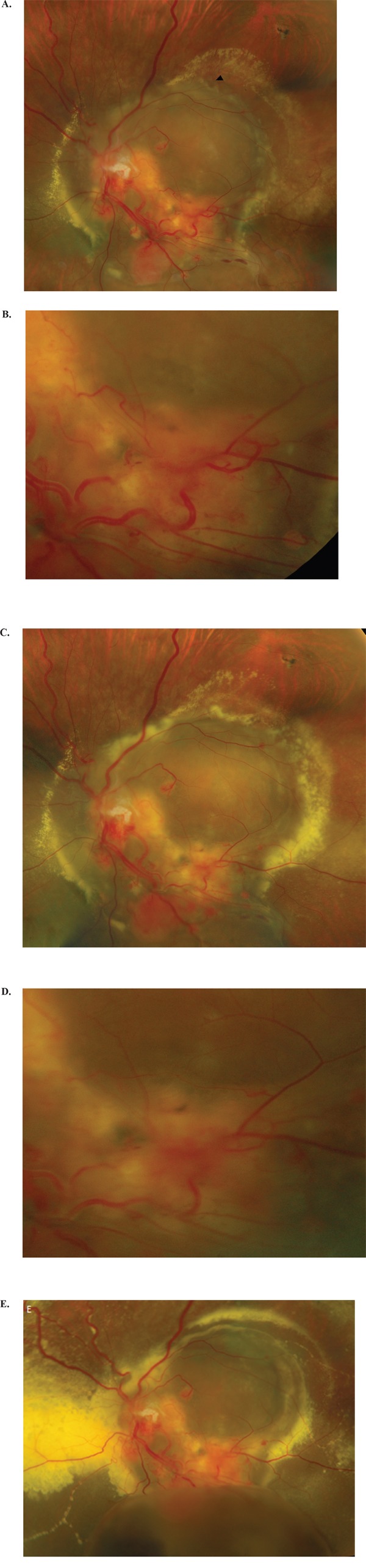
Images of retinal capillary hemangioblastomas (RCH) in the left eye of a 31-year-old patient **A.** Before treatment; multiple RCH associated with serous retinal detachment (SRD) occupying the entire posterior pole, with accumulation of hard exudates on the outskirts of the image; the presence of several papillary hemangioblastomas is also evident; the lower temporal vessels are dilated and drain several large RCH. **B.** RCH detail. **C & D.** One week after initiation of sunitinib, showing increased retinal exudates (C) and the lower temporal vessels appear less dilated (D). The size of the RCH is stable or slightly increased. **E.** Sunitinib was stopped after 16 days; this image is taken 2 weeks later, showing increased retinal exudates (particularly temporal to the optic nerve) and serous retinal detachment with appearance of a bulky lower pocket.

Two patients showed a transitory clinical improvement to sunitinib and one had a long-lasting response. The first patient was a young female with bilateral retinal hemangioblastomas and serous retinal detachment (SRD). This was a form with multiple retinal capillary hemangioblastomas (RCH) posterior pole causing significant exudation with a big macular SRD bubble (Figure 1A). RCH were not accessible for laser treatment or otherwise, and the patient was treated with sunitinib. The patient had limited vision at baseline, with no evidence of hemorrhage (Figure 1). After 10 days of sunitinib 50 mg/day, fundus examination revealed decreased RCH vascularization. The first control seemed positive in that there was a decrease in the caliber of lower temporal vessels (Figure 1C). A decrease in the diameter of the artery which vascularizes the retinal hemangioblastoma upon laser photocoagulation suggested a decrease in blood flow; the observed coagulation of the RCH is a sign of sunitinib effectiveness. The RCH located below the lower vascular branch appeared to have increased in size, along with the size and count of cerebellar and medullary hemangioblastomas which increased later during cycle 1. The cycle 2 starting dosage was 37.5 mg/day, reduced to 25 mg/day after 2 weeks for poor tolerability. Renal lesions decreased to a level seen 3 months previously. After the 2-week break prior to cycle 3, vision was worse than at baseline. The patient underwent additional surgery and was withdrawn due to lack of clinical improvement and toxicity. Verification carried out two weeks after premature discontinuation of sunitinib showed significantly increased exudation. However, care must be taken when interpreting this case. The fundus generally evolved to a worsened state despite sunitinib, although the decrease in the caliber of these lower temporal vessels was initially encouraging. It is possible that the fundus would have improved if cycle 1 had been completed, but what we actually saw was a worsening at discontinuation of cycle 1 that continued despite a second treatment cycle.

**Figure 2:**
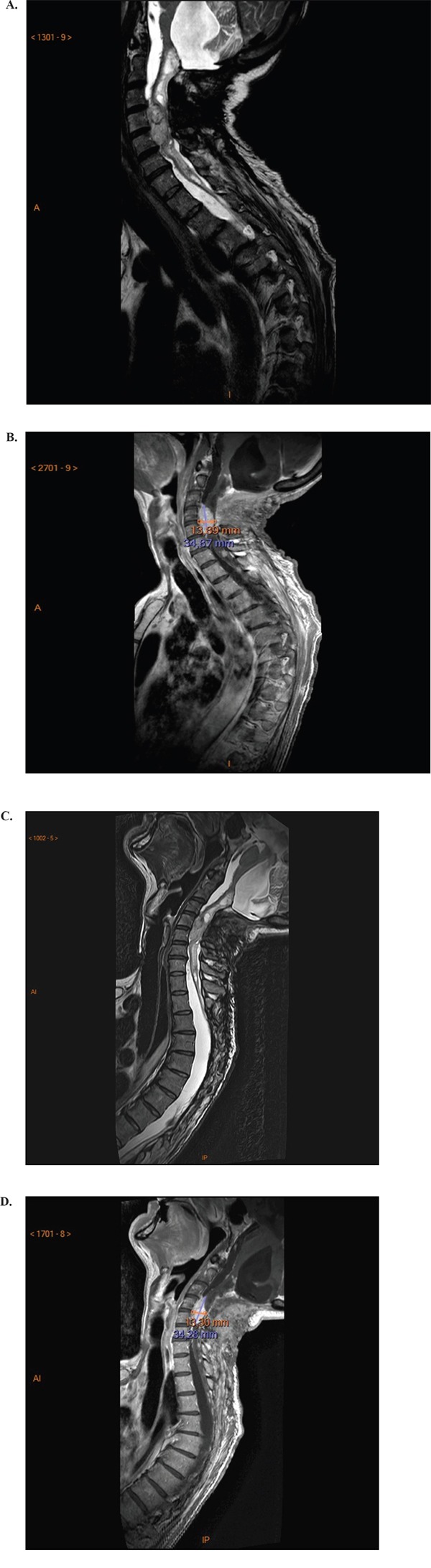
Magnetic resonance imaging showing a large hemangioblastoma at the spine cervical level at baseline **(A & B)**; and no change in hemangioblastoma size after 4 weeks’ sunitinib **(C & D)**.

The second patient was a male paraplegic presenting with a medullar hemangioblastoma on MRI at baseline (35x14mm; Figure 2A&2B). This patient experienced a good response during cycle 1, with return of mobility and the ability to use his computer mouse; there was no significant medullar decompression on MRI after 4 weeks’ sunitinib; this could explain the clinical improvement (Figure 2C & 2D). During cycle 2 (sunitinib 37.5 mg/day), clinical improvement was less and that patient was unable to move his hand; he was hospitalized due to complications.

The third patient was a young male in good general condition with multiple neuraxis hemangioblastomas. He received sunitinib 50 mg/day (schedule 4/2) for 1 year with poor tolerability and recurrent incapacitating diarrhea. No MRI changes were observed and treatment was discontinued. One month later the patient showed clinical improvement with resolving toxicity.

### Overall survival

No deaths occurred during sunitinib treatment. One patient died after 5 months’ follow-up due to disease progression. Median survival could not be calculated due to a lack of events.

### Toxicity

The total number of treatment-emergent adverse events in the five study patients was 137; fatigue, mucositis, diarrhea and nausea were the most common events, with ≥4 events of each. The majorities of adverse events were grade 1-2 in severity and were consistent with the expected tolerability profile of sunitinib. Most adverse events recurred from once cycle to the next without any important change in severity, indicating good management of toxicity with strategies such as dose reduction. Grade 3/4 adverse events occurring in 1 patient each were fatigue, diarrhea, stomatitis, dysphagia, dyspnea, headache, hydrocephalia, thrombocytopenia, acute respiratory distress syndrome, hemorrhoids, interstitial pneumonia, retinal detachment and sepsis.

### Discontinuations

Reasons for discontinuing sunitinib therapy were disease progression (n=1), unacceptable toxicity (n=3) and lack of clinical improvement after 7 cycles (10.5 months) with unacceptable toxicity (n=1).

## DISCUSSION

Our experience suggests that it is very difficult to recruit patients with VHL disease in a prospective clinical trial, even when facilitated by the PREDIR network. The study results showed that sunitinib may have some efficacy in naïve-patients with advanced VHL disease but treatment may be poorly tolerated by this frail patient population. We observed significant inter-patient variability in the response to sunitinib, and noted that treatment was more active against mRCC tumors than other manifestations of VHL disease.

The only other clinical trial of sunitinib in VHL disease also showed differential effects of treatment (i.e. good for renal tumor lesions and little or no activity against hemangioblastomas) [13]. This pattern was also seen in a case report and a retrospective case review of VHL disease patients who had responses in mRCC tumors, but not other VHL lesions, after sunitinib [11, 14]. In addition, prolonged partial responses to sunitinib were reported for mRCC lesions in a series of four VHL disease cases [15]. A potential explanation for this difference in tissue responsiveness for RCC versus other tumors appears to be related to greater expression of VEGF receptor in RCC tumors compared with hemangioblastomas (in which the fibroblast growth factor pathway might be relatively more important) [13].

The hemangioblastoma component of VHL disease does appear to be more difficult to treat. An earlier study with semaxanib showed disease stabilization in 33% of patients with hemangioblastomas [9], but patient numbers were small and this agent is no longer being actively developed. Some regression of VHL-related hemangioblastomas has been reported in individual patients treated with pazopanib [16] or bevacizumab [17], whereas ranibizumab had little benefit in a small study of patients with VHL-related retinal hemangioblastoma [18]. The overall lack of efficacy observed on hemangioblastomas on MRI suggests that sunitinib, and probably other similar agents, have poor activity against cystic lesions. On the other hand, two patients with CNS hemangioblastomas in our study had marked, transient responses to sunitinib. Whether or not this constitutes a signal to be explored for this disease is unknown. Such subjective improvements in the absence of any objective response to sunitinib therapy, although usually brief, do not appear to have been specifically reported previously.

The nature of treatment-emergent adverse events was similar in our study and the previous clinical trial [13], with fatigue, mucositis and diarrhea being the most common toxic effects. Although the majority of adverse events in our study were grade 1-2 in severity, two serious events were reported. In addition, sunitinib dosage reductions were required in three out of five patients to manage emerging toxicity. In the first clinical study with sunitinib in VHL disease, treatment was discontinued for toxicity (neutropenia) in one of the fifteen patients included [13]. The tolerability profile of sunitinib was better in a series of four patients with VHL-related RCC, with only one patient requiring dose modification due to side effects [15]. Differences between our study and previous reports [13, 15] may be due to differences in age and disease severity between the populations. Based on these small comparisons it is possible that sunitinib treatment-related toxicity might be greater in older patients and/or those with more severe VHL disease. Higher disease severity could also have been a factor contributing to the lower effectiveness of sunitinib in our study compared with the previous phase II clinical trial [13].

An important limitation of our study is the small number of patients. However, VHL disease is relatively rare, making identification of larger patient groups difficult, even within the PREDIR network. The first phase II published study was stopped early for low accrual and enrolled 15 patients over a 3-year recruitment period [13]. This equates to an average of 5 patients per year, similar to the number of patients eligible for our study over nearly 1 year (n=7). Heterogeneity of the patient population is another issue in VHL disease studies, including this one, but represents the profile of patients that would be encountered in clinical practice.

In conclusion, our data suggest that sunitinib is of limited benefit in patients with advanced VHL disease and that treatment-related toxicity is an important limiting factor. Sunitinib appeared to be a more appropriate treatment option for VHL-related RCC than for other VHL-related lesions.

## MATERIALS AND METHODS

### Study design

This multicenter study, phase II, open-label study from the PREDIR network was approved by the local ethics committee and French Health authorities. This study is registered with ClinicalTrials.gov (NCT01168440). The trial was conducted in accordance with Good Clinical Practice and the Declaration of Helsinki. All patients provided written informed consent prior to study participation.

### Patient population

Consecutive patients presenting between 3 May 2010 and 28 March 2011 were first screened by an oncogenetics specialist to confirm the diagnosis of VHL. Patients with genetically- or clinically-confirmed VHL disease and related symptoms that were refractory to conventional treatment were then referred to a medical oncologist to evaluate eligibility. To be eligible, patients had to have at least one of the following lesions: retinal hemangioblastoma that could no longer be treated with laser therapy or cryotherapy associated with progressive loss of vision; cerebellar, bulbar, spinal, or cerebellopontine angle hemangioblastoma or endolymphatic sac tumor causing neurological symptoms that was not amenable to further surgery or had recurred after a first surgery; multiple or bilateral tumors not accessible to conservative surgery or tumors that had recurred after surgery and/or radiofrequency ablation, or advanced/metastatic RCC; inextirpable or advanced neuroendocrine pancreatic tumors. Other inclusion criteria were age ≥18 years, European Co-operative Oncology Group Performance Status (ECOG-PS) 0-2, life expectancy ≥3 months, and biological and clinical values within normal limits. Patients who had received previous treatments for VHL (surgery, chemotherapy, radiotherapy) could be included if these had been completed >4 weeks prior to the study. Previously radiated lesions were considered as target lesions only if they demonstrated unequivocal evidence of growth on imaging.

### Treatment

Oral sunitinib 50 mg/day was given for 28 days followed by a 2-week rest period (schedule 4/2; overall 6-week treatment course) for a maximum of 8 courses. Treatment was continued until disease progression, unacceptable toxicity, withdrawal of patient consent, or investigator/sponsor decision to discontinue the patient's participation. Dose interruption or reduction was permitted at the investigator's discretion if required to ensure patient safety. The sunitinib dosage could be reduced to 37.5 mg/day then to 25 mg/day, with the same 4 week on/2 weeks off dosing schedule. Treatment was discontinued if toxicity had not resolved to acceptable levels within 4 weeks. Patients who were responding well and tolerating therapy at the end of the study could continue sunitinib therapy at the investigator's discretion.

### Clinical evaluations

Physical examination, ECOG-PS, body weight, vital signs, laboratory parameters, and toxicity were assessed at baseline, during the 2-week rest period at the end of each cycle, and 28 days after the end of treatment. Baseline and follow-up evaluations of target lesions were performed during the rest period at the end of cycles 1 and 2 and then every cycle for retinal hemangioblastoma and at the end of every second cycle for all other tumors. Tumor assessment consisted of magnetic resonance imaging (MRI) for CNS lesions, computed tomographic (CT) scan or MRI or echography for abdominal lesions, direct ophthalmoscopy, using fluorescein angiography with photographs, color testing, and visual field testing for retinal lesions, CT scan and echography for renal tumors (plus a contrast-enhanced ultrasound on day 15 of cycle 1 to assess possible early response). Responses were classified according to RECIST criteria v1.0. Toxicity was assessed according to NCI-CTC v3.0 criteria in all patients who received ≥1 dose of sunitinib.

### Endpoints

The primary endpoint was objective response rate. Secondary endpoints included the safety and tolerability of sunitinib, and time-to-event endpoints (time to disease progression, progression-free survival, time to response, duration of response and overall survival).

### Statistical analysis

Data were collected by each investigator from patient medical records at their own institution and sent to the coordinating site using an electronic case report form (CRF) designed for this study. Patients were identified in the CRF using country, initials and birth date. Data management was performed and queries sent back to investigators for additional clarification, if needed. Patients with unrecoverable missing key data (histology, treatments lines, treatment start and end dates, CT scan dates, objective response) were excluded. The intention-to-treat population (ITT) and s*afety population* included patients who signed informed consent and/or received ≥1 sunitinib dose. Analyses were performed using XLStat (ADinsoft).

## References

[1] Pal SK, Nelson RA, Vogelzang N (2013). Disease-specific survival in de novo metastatic renal cell carcinoma in the cytokine and targeted therapy era. PLoS One.

[2] Hudes G, Carducci M, Tomczak P, Dutcher J, Figlin R, Kapoor A, Staroslawska E, Sosman J, McDermott D, Bodrogi I, Kovacevic Z, Lesovoy V, Schmidt-Wolf IG (2007). Temsirolimus, interferon alfa, or both for advanced renal-cell carcinoma. N Engl J Med.

[3] Raymond E, Dahan L, Raoul JL, Bang YJ, Borbath I, Lombard-Bohas C, Valle J, Metrakos P, Smith D, Vinik A, Chen JS, Horsch D, Hammel P (2011). Sunitinib malate for the treatment of pancreatic neuroendocrine tumors. N Engl J Med.

[4] Yao JC, Shah MH, Ito T, Bohas CL, Wolin EM, Van Cutsem E, Hobday TJ, Okusaka T, Capdevila J, de Vries EG, Tomassetti P, Pavel ME, Hoosen S (2011). Everolimus for advanced pancreatic neuroendocrine tumors. N Engl J Med.

[5] Keutgen XM, Hammel P, Choyke PL, Libutti SK, Jonasch E, Kebebew E (2016). Evaluation and management of pancreatic lesions in patients with von Hippel-Lindau disease. Nat Rev Clin Oncol.

[6] Motzer RJ, Hutson TE, Tomczak P, Michaelson MD, Bukowski RM, Oudard S, Negrier S, Szczylik C, Pili R, Bjarnason GA, Garcia-del-Muro X, Sosman JA, Solska E (2009). Overall survival, updated results for sunitinib compared with interferon alfa in patients with metastatic renal cell carcinoma. J Clin Oncol.

[7] Girmens JF, Erginay A, Massin P, Scigalla P, Gaudric A, Richard S (2003). Treatment of von Hippel-Lindau retinal hemangioblastoma by the vascular endothelial growth factor receptor inhibitor SU5416 is more effective for associated macular edema than for hemangioblastomas. Am J Ophthalmol.

[8] Jennens RR, Rosenthal MA, Lindeman GJ, Michael M (2004). Complete radiological and metabolic response of metastatic renal cell carcinoma to SU5416 (semaxanib) in a patient with probable von Hippel-Lindau syndrome. Urol Oncol.

[9] Madhusudan S, Deplanque G, Braybrooke JP, Cattell E, Taylor M, Price P, Tsaloumas MD, Moore N, Huson SM, Adams C, Frith P, Scigalla P, Harris AL (2004). Antiangiogenic therapy for von Hippel-Lindau disease. JAMA.

[10] Jimenez C, Cabanillas ME, Santarpia L, Jonasch E, Kyle KL, Lano EA, Matin SF, Nunez RF, Perrier ND, Phan A, Rich TA, Shah B, Williams MD (2009). Use of the tyrosine kinase inhibitor sunitinib in a patient with von Hippel-Lindau disease: targeting angiogenic factors in pheochromocytoma and other von Hippel-Lindau disease-related tumors. J Clin Endocrinol Metab.

[11] Tsimafeyeu I (2015). Sunitinib treatment of metastatic renal cell carcinoma in von Hippel-Lindau disease. J Cancer Res Ther.

[12] Kobayashi A, Takahashi M, Imai H, Akiyama S, Sugiyama S, Komine K, Saijo K, Takahashi M, Takahashi S, Shirota H, Sato N, Fujishima F, Shuin T (2016). Attainment of a Long-term Favorable Outcome by Sunitinib Treatment for Pancreatic Neuroendocrine Tumor and Renal Cell Carcinoma Associated with von Hippel-Lindau Disease. Intern Med.

[13] Jonasch E, McCutcheon IE, Waguespack SG, Wen S, Davis DW, Smith LA, Tannir NM, Gombos DS, Fuller GN, Matin SF (2011). Pilot trial of sunitinib therapy in patients with von Hippel-Lindau disease. Ann Oncol.

[14] Roma A, Maruzzo M, Basso U, Brunello A, Zamarchi R, Bezzon E, Pomerri F, Zovato S, Opocher G, Zagonel V (2015). First-Line sunitinib in patients with renal cell carcinoma (RCC) in von Hippel-Lindau (VHL) disease: clinical outcome and patterns of radiological response. Fam Cancer.

[15] Kim HC, Lee JS, Kim SH, So HS, Woo CY, Lee JL (2013). Sunitinib treatment for metastatic renal cell carcinoma in patients with von hippel-lindau disease. Cancer Res Treat.

[16] Kim BY, Jonasch E, McCutcheon IE (2012). Pazopanib therapy for cerebellar hemangioblastomas in von Hippel-Lindau disease: case report. Target Oncol.

[17] Hrisomalos FN, Maturi RK, Pata V (2010). Long-term use of intravitreal bevacizumab (Avastin) for the treatment of von hippel-lindau associated retinal hemangioblastomas. Open Ophthalmol J.

[18] Wong WT, Liang KJ, Hammel K, Coleman HR, Chew EY (2008). Intravitreal ranibizumab therapy for retinal capillary hemangioblastoma related to von Hippel-Lindau disease. Ophthalmology.

